# Clinical Predictors and Accuracy of Empiric Tuberculosis Treatment among Sputum Smear-Negative HIV-Infected Adult TB Suspects in Uganda

**DOI:** 10.1371/journal.pone.0074023

**Published:** 2013-09-06

**Authors:** Lydia Nakiyingi, John Mark Bwanika, Bruce Kirenga, Damalie Nakanjako, Catherine Katabira, Gloria Lubega, Joseph Sempa, Barnabas Nyesiga, Heidi Albert, Yukari C. Manabe

**Affiliations:** 1 Infectious Diseases Institute, Makerere College of Health Sciences, Kampala, Uganda; 2 School of Medicine, Makerere University College of Health Sciences, Kampala, Uganda; 3 Foundation for Innovative New Diagnostics (FIND), Kampala, Uganda; 4 Division of Infectious Diseases, Department of Medicine, Johns Hopkins University, Baltimore, Maryland, United States of America; Institut de Pharmacologie et de Biologie Structurale, France

## Abstract

**Introduction:**

The existing diagnostic algorithms for sputum smear-negative tuberculosis (TB) are complicated, time-consuming, and often difficult to implement. The decision to initiate TB treatment in resource-limited countries is often largely based on clinical predictors. We sought to determine the clinical predictors and accuracy of empiric TB treatment initiation in HIV-infected sputum smear-negative TB suspects using sputum culture as a reference standard.

**Setting:**

Out-patient HIV-TB integrated urban clinic in Kampala, Uganda.

**Methods:**

HIV-infected TB suspects were screened using sputum smear microscopy, and mycobacterial sputum liquid and solid cultures were performed. Smear results were made available to the clinician who made a clinical decision on empiric TB treatment initiation for sputum smear-negative patients. Clinic records were reviewed for patients whose sputum smears were negative to collect data on socio-demographics, TB symptomatology, chest X-ray findings, CD4 cell counts and TB treatment initiation.

**Results:**

Of 253 smear-negative TB suspects, 56% (142/253) were females, median age 38 IQR (31–44) years, with a median CD4 cell count of 291 IQR (150–482) cells/mm^3^. Of the 85 (33.6%) smear-negative patients empirically initiated on TB treatment, 35.3% (n = 30) were sputum culture positive compared to only 18 (10.7%) of the 168 untreated patients (p<0.001). Abnormal chest X-ray [aOR 10.18, 95% CI (3.14–33.00), p<0.001] and advanced HIV clinical stage [aOR 3.92, 95% CI (1.20–12.85), p = 0.024] were significantly associated with empiric TB treatment initiation. The sensitivity and specificity of empiric TB treatment initiation in the diagnosis of TB in HIV-infected patients after negative smear microscopy was 62.5% and 73.7% respectively.

**Conclusion:**

In resource-limited settings, clinically advanced HIV and abnormal chest X-ray significantly predict a clinical decision to empirically initiate TB treatment in smear-negative HIV-infected patients. Empiric TB treatment initiation correlates poorly with TB cultures. Affordable, accurate and rapid point-of-care diagnostics are needed in resource-limited settings to more accurately determine which HIV-infected TB suspects have smear-negative TB.

## Introduction

Tuberculosis (TB) is a major cause of death among HIV-infected patients [1], [2], [3], [4], [5], [6]. HIV infection increases the risk of both primary active TB disease and reactivation. Eighty percent of the TB-HIV co-infected cases worldwide occur in sub-Saharan Africa (SSA) [2], [7]. Uganda ranks 19^th^ among the world’s 22 high-burden TB countries with an estimated incidence rate of 193 cases per 100,000 population. Among new TB patients, 53% are HIV-positive [7]. Early identification of TB is essential for prompt initiation of therapy to reduce TB-associated morbidity and mortality as well as transmission.

Many countries in SSA continue to rely on sputum smear microscopy for the diagnosis of pulmonary TB despite the HIV epidemic which has changed the presentation of TB and reduced the sensitivity of smear microscopy (2–3). Several studies in SSA have found an increased prevalence of sputum smear-negative (SSN) TB in HIV-infected patients [6], [8], [9], [10], [11]. This has emphasized the need for more sensitive and affordable diagnostic tools [6], [9], [12], [13], [14]. However, there are few alternatives to the existing smear microscopy in resource-limited settings (RLS) which are inexpensive, easy to use and widely available [6], [12], [13], [15].

Although diagnostic algorithms for SSN TB exist, these require 11 to 34 days to establish a diagnosis [16], [17] during which time the disease could progress and patients could be lost to care. Furthermore, liquid and solid culture technology is not widely available due to technical and biosafety requirements. Therefore, clinicians in RLS often make diagnoses largely based on clinical predictors [18], [19] which may result in under- or over-diagnosis. Moreover, these clinical predictors are mostly subjective; with the criteria being determined by the treating physician.

There is limited data on the clinical criteria commonly used by treating physicians in RLS to empirically initiate TB treatment in HIV-infected SSN TB suspects and the accuracy of this clinical decision when mycobacterial sputum cultures are used as the reference test. We aimed to determine the clinical predictors and accuracy of empiric TB treatment initiation in HIV-infected sputum smear-negative TB suspects using sputum culture as a reference standard. Findings from this study will provide potentially important information to clinicians in RLS that may improve their approach to clinical diagnosis of SSN TB in HIV-infected patients.

### Ethics Statement

The study was part of a fluorescence microscopy field evaluation research project that was approved by the Scientific Review Board of the Infectious Diseases Institute (IDI), the Institutional Review Board (IRB) of Makerere University and the Uganda National Council for Science and Technology. The use of routinely collected clinical data at the IDI was approved by the Scientific Review Board of the Infectious Diseases Institute, the IRB of Makerere University and the Uganda National Council for Science and Technology. Patients are not consented since the data are primarily used for clinical care and no personal identifiers are seen by the researchers. This process was approved by the above mentioned review boards.

## Methods

### Study Setting and Design

The study was conducted at the Adult Infectious Diseases Clinic (AIDC) of the Infectious Diseases Institute (IDI), at the Makerere University College of Health Sciences in Kampala, Uganda. The AIDC is a center of excellence that provides outpatient care to over 10,000 HIV-positive patients. HIV-infected patients with clinically suspected TB during routine medical interactions at the AIDC are transferred to an integrated TB/HIV clinic [20] where they are evaluated by a TB clinic doctor who may request investigations for TB. Available investigations for pulmonary TB include fluorescence smear microscopy and chest X-ray (CXR); extra pulmonary TB investigations are ordered according to the suspected site of the disease. No mycobacterial culture facilities are available for routine evaluation. Patients diagnosed with active TB are treated in accordance with the existing treatment guidelines from the Uganda Ministry of Health National TB program [21]. All TB investigations and treatment at the AIDC are free of charge. Patients requiring in-patient care are referred to Mulago National Referral Hospital, a tertiary care hospital in the same complex.

### Patient Recruitment

Between June 2009 and January 2010, HIV-infected TB suspects [as per the WHO intensified case finding guidelines [22]] aged ≥18 years were screened for TB using fluorescence microscopy (FM) and Ziehl-Neelsen (ZN) smear microscopy performed on two spot sputum samples as part of a FM field evaluation [23]. Additionally, mycobacterial growth indicator tube (MGIT) and Lowenstein-Jensen (LJ) cultures were performed on the same sputum samples. Sputum smear and chest X-ray results were made available to the clinician who decided on TB treatment initiation. MGIT and LJ culture results were also made available to the treating clinician when results were obtained. The average turnaround time for MGIT and LJ cultures was three weeks and four weeks respectively and therefore these results did not influence the clinical decision on empiric TB treatment.

We retrospectively reviewed clinic records for patients whose sputum smears were negative on both ZN and FM microscopy (sputum smear-negative TB suspects) to collect data on the following: clinician’s decision on empirical TB treatment initiation, socio-demographics (age, sex), clinical data (antiretroviral therapy (ART) history, previous TB treatment, signs and symptoms at presentation, respiratory examination findings), chest X-ray (CXR) and CD4 cell counts.

### Laboratory Procedures

After collection, specimens were kept in a cool box until they were transferred to a refrigerator upon receipt in the laboratory at the IDI. Smears were prepared from each specimen and stained using Auramine-O method. The rest of the sputum sample was transported to the Foundation for Innovative New Diagnostics (FIND) TB research laboratory situated at the National TB Reference laboratory where MGIT and LJ culture were performed according to the standard methods with Capilia™ TB test used for *Mycobacterium tuberculosis* (MTB) complex identification [23]. Additionally, direct and concentrated ZN smear microscopy was performed at the FIND laboratory.

### Culture and Identification

As previously described [23], sputum was decontaminated by standard NALC-NaOH procedure (1.5% NaOH final concentration and 166% PANTA concentration). Following neutralization and centrifugation, the pellet was suspended in 1 ml phosphate buffer pH 6.8. Approximately 0.5 ml was used to inoculate MGIT culture and 0.1 ml to inoculate LJ culture. Positive cultures were identified using Capilia TB –Neoassay (Tauns Laboratories Inc. Namazu, Japan). Capilia TB- Neo negative isolates were tested using Genotype CM assay (Hain Life Science, Pehren, Germany).

### Data Management and Statistical Analysis

Clinical data from the IDI clinic records were merged with the laboratory data, both of which were captured using Microsoft Access 2003 and later exported into STATA version 11.2 which was used for analysis.

The primary outcome was the clinical decision to empirically initiate TB treatment in a SSN HIV-infected TB suspect. Continuous variables were summarized using medians and inter-quartile ranges (IQR) while categorical variables were summarized using frequencies and percentages.

Using Wilcoxon rank sum test for continuous variables and either chi-square test or Fisher’s Exact test for categorical variables, we compared the characteristics of the study population by the strata of the primary outcome.

To identify predictors of empiric TB treatment initiation among SSN HIV-infected TB suspects, bivariate and multivariate logistic regression models were built and results presented as unadjusted and adjusted Odds ratio (OR) with 95% confidence interval (95% CI) respectively.

The modeling process involved selecting all factors from bivariate analysis that had a P-value of ≤0.2 and those of known clinical significance, specifically previous TB treatment and taking antiretroviral therapy for inclusion in the initial multivariate logistic regression model.

Using a systematic backward approach, non-significant variables were removed from the model until no further variables were eligible for removal to arrive at the final parsimonious model. A P-value of <0.05 in the final model was considered statistically significant.

To determine the accuracy of empirical TB treatment initiation in the diagnosis of TB in SSN HIV-infected patients, a two-by-two table was used to calculate sensitivity, specificity, positive and negative predictive values using either LJ or MGIT sputum culture positivity as the reference standard.

## Results

**Figure 1:**
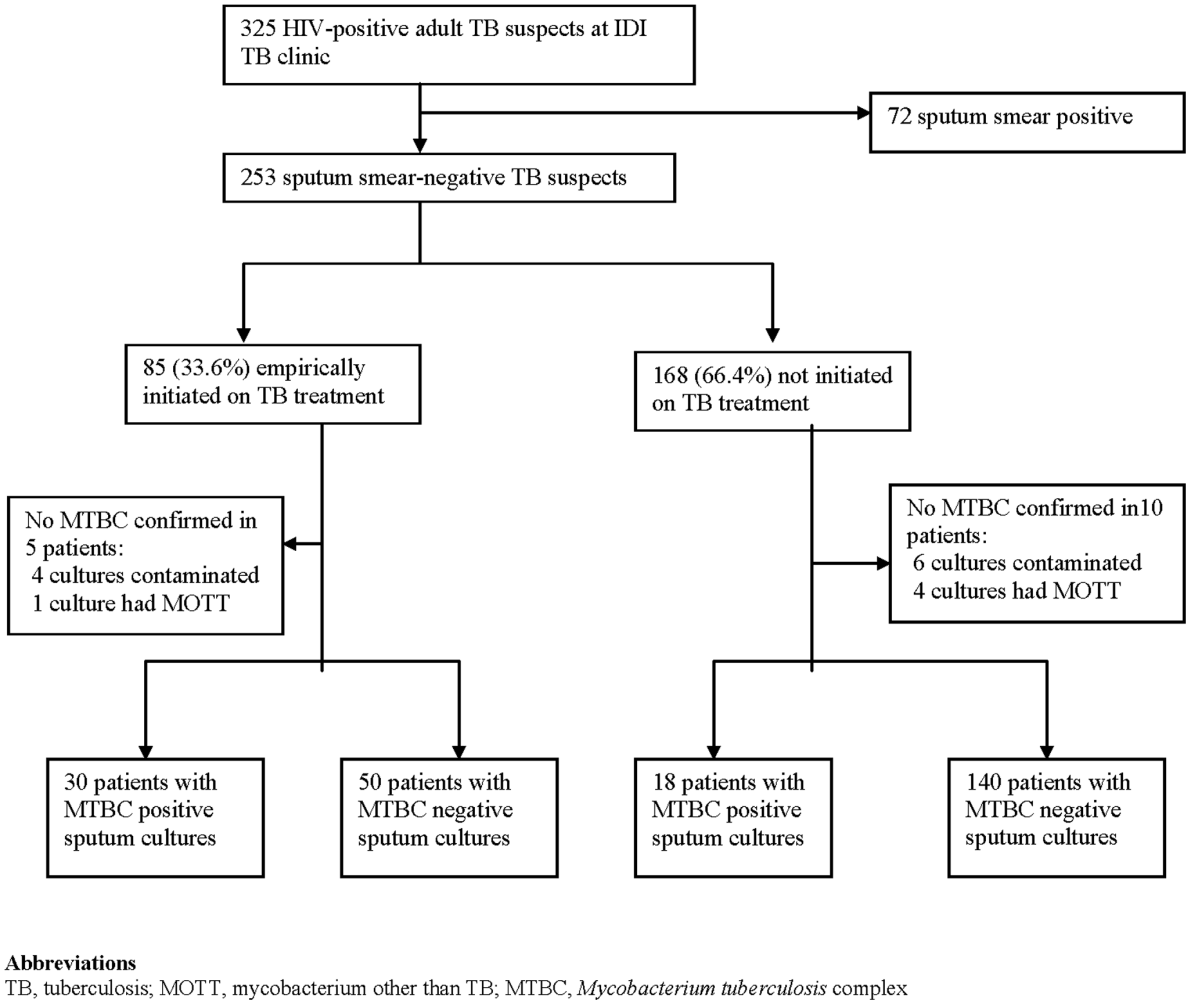
Patient enrollment flow diagram showing the number of patients enrolled and analyzed as well as the TB culture results distribution.

Of the 325 HIV-infected patients who provided sputum samples for microscopy, 253 (77.8%) were SSN and were therefore eligible for this analysis **(** **Figure 1** **).** The other 72 (22.2%) had positive sputum smears. Of the 253 SSN patients, 56% (142/253) were females; the median age was 38 (IQR 31–44) years and median CD4 291 (IQR 150–482) cells/mm^3^ **(** **Table 1** **)**.

**Table 1:** Comparing characteristics of sputum smear-negative study participants empirically initiated on TB treatment and those who were not treated.

Demographic and clinical Characteristic	Total, N = 253	Treated for TB,N = 85	Not treated forTB,N = 168	P value
Median age, years (IQR)	38 (31–44)	36 (31–41)	38 (32–45)	0.141
Median Weight, KG (IQR)	**56 (49–63)**	**51 (48–55)**	**57 (50–64)**	**0.014**
Female sex, # (%)	142 (56.1)	45 (52.9))	97 (58.1))	0.437
Median CD4 (IQR) (cells/mm^3^)	291 (150–482)	283 (130–398)	302 (157–511)	0.185
*B symptoms present, (fever, weight loss, excessnight sweats) (N = 215)	**174 (80.9)**	**70 (90.3)**	**104 (75.9)**	**0.016**
*Shortness of breath, (N = 214)	60 (28.0)	27 (34.6)	33 (24.3)	0.106
*Chest pain present, (N = 216)	113 (52.3)	42 (53.9)	71 (51.5)	0.735
*Abnormal respiratory exam, (N = 182)	37 (20.3)	16 (25.8)	21 (17.5)	0.189
*Abnormal CXR, # (%) (N = 131)	**96/131 (73.3)**	**58/64 (90.6)**	**38/67 (53.7)**	**<0.001**
Previous TB treatment	25 (9.9)	11 (12.9)	14 (8.3)	0.250
*WHO stage, (N = 231)Stage 3 and 4	**113 (48.9)**	**48 (59.3)**	**65 (43.3)**	**0.022**
*On ART medication, N = 241,	162 (67.2)	56 (65.9)	106 (68.0)	0.744

* means the variable number is less than the total N = 253. These were missing data in the clinic patients records most of which were not recorded by the treating clinicians.

**Abbreviations**: IQR, Interquatile range; CXR, chest X-ray; TB, tuberculosis; WHO, World Health Organization; ART, Antiretroviral therapy.

Of the 253 SSN patients, 85/253 (33.6%) were empirically initiated on TB treatment. Of these 85 empirically treated patients, 30 (35.3%) were culture-positive for MTB complex (MTBC) while only 18 (10.7%) of the 168 who were not treated were culture-positive **(** **Figure 1** **)**.There was a statistically significant difference in culture positivity between the two groups (p<0.001). The median body weight was significantly lower in the empirically treated group when compared to the untreated group; 51 Kg (IQR 48–55) in the treated group versus 57 Kg (IQR 50–64) in the untreated group [OR 0.93, 95% CI (0.88–0.99), p = 0.014] (**Table 1**). There was no significant difference in median age and median CD4 cell count between the group that was empirically initiated on TB treatment and the group that was not.

### Clinical Predictors of Empiric TB Treatment Initiation

Constitutional TB symptoms (also known as B symptoms) including fever, weight loss of >10% in four weeks, excess night sweats [OR 2.78, 95% CI (1.21–6.37), p = 0.016]; clinically advanced HIV (WHO stages 3 and 4) [OR 1.90, 95% CI (1.10–3.29), p = 0.022]; and abnormal chest radiological features [OR 7.38, 95% CI (2.80–19.45), p<0.001] were significantly more common in the group that was empirically initiated on TB treatment compared to the group that was not **(** **Table 2** **).** On multivariate analysis, abnormal CXR [aOR 10.18, 95% CI (3.14–33.00), p<0.001] and advanced HIV WHO clinical stage 3 or 4 [aOR 3.92, 95% CI (1.20–12.85), p = 0.024] remained significantly associated with empiric TB treatment initiation.

**Table 2:** Multivariate analysis for predictors of empiric TB treatment in sputum smear-negative HIV-infected TB suspects*.

Demographic and clinical Characteristic	OR (95%CI)	aOR (95%CI)	P value
Age (years)	0.98 (0.95–1.01)	–	–
Body weight (Kg)	0.93 (0.88–0.99)	–	–
Female sex	1.23 (0.73–2.08)	–	–
CD4 cell count (cells/mm^3^ per 50 cells increase)	0.96 (0.91–1.02)	–	–
B symptoms present	**2.78 (1.21–6.37)**	2.56 (0.76–8.68)	0.131
Shortness of breath	1.65 (0.90–3.04)	1.61 (0.67–3.85)	0.283
Chest pain present	1.10 (0.44–1.49)	–	–
Abnormal respiratory exam	1.64 (0.78–3.43)	–	–
Abnormal CXR	**7.38 (2.80–19.45)**	**10.18 (3.14–33.00)**	**<0.001**
Previous TB treatment	1.63 (0.71–3.77)	–	–
WHO stage 3 and 4	**1.90 (1.10–3.29)**	**3.92 (1.20–12.85)**	**0.024**
On ART medication	0.91 (0.52–1.60)	0.34 (0.09–1.25)	0.105

* N = 123 with complete records. The model adjusted for B symptoms, shortness of breath, CXR findings, ART therapy, WHO clinical stage.

**Abbreviations**: CXR, chest X-ray; TB, tuberculosis; WHO, World Health Organization; ART, Antiretroviral therapy; OR, Odds ratio; aOR, adjusted odds ratio; CI, Confidence intervals.

In a sensitivity analysis eliminating patients who had missing clinical information, the clinical predictors remained the same (data not shown).

### Mycobacterial Sputum Cultures

Of the 253 SSN HIV-infected TB suspects; 20.9% (53/253) cultures grew mycobacteria, of which 90.6% (48/53) were MTBC and 9.4% (5/53) were mycobacteria other than TB (MOTT); 75.1% (190/253) were MTB culture negative while 4% (10/253) were contaminated **(** **Table 3** **).**

**Table 3:** Distribution of sputum TB culture results.

Mycobacterial cultureresults	Total,N = 253	Treated for TB,N = 85	Not treated for TB,N = 168	P value
Positive for MTBC, n (%)	48 (19)	30(35.3)	18(10.7)	<0.001
Negative, n (%)	190 (75)	50(58.9)	140(83.3)	
Positive for MOTT, n (%)	5(2)	1(1.2)	4(2.3)	
Contaminated, n (%)	10(4)	4(4.7)	6(3.4)	

Abbreviations: MTBC, *Mycobacterium tuberculosis* complex; MOTT, mycobacteria other than tuberculosis; TB, tuberculosis.

### Accuracy of Empiric TB Treatment Initiation in the Diagnosis of TB in SSN HIV-infected TB Suspects

Participants whose sputum cultures were contaminated (n = 10) or positive for MOTT (n = 5) were excluded from the accuracy analysis. Of the remaining 238 participants with corresponding clinical diagnosis and TB culture results, 48/238 (20.2%) were MTBC culture positive.

Of the 48 MTBC culture positive patients, 30 (62.5%) were correctly diagnosed and treated for TB on the basis of clinical judgment while 140 (73.7%) of the 190 MTBC culture negatives were correctly clinically judged as no TB and therefore not treated. The sensitivity and specificity of clinical judgment in diagnosis of TB in SSN HIV-infected patients was 62.5% and 73.7% respectively. The positive and negative predictive values were 37.5% and 88.6% respectively.

### Characteristics of Culture Positive-untreated and Culture Negative-treated

Of the 18 participants who were culture positive but not empirically initiated on TB treatment; only 66.6% had constitutional symptoms, 78.6% had abnormal CXR, 38.1% were WHO clinical stage 3 & 4, and 76% were on ART. On the other hand, the majority of the 50 participants who were culture negative but empirically treated had constitutional symptoms (86.4%), abnormal CXR (89.5%) and only 54% were on ART.

All MTBC culture positive patients were eventually treated for TB when MTB culture results were made available to the treating clinicians. However, the effect of TB treatment delays on the participants whose TB treatment was initiated after culture results were not assessed.

## Discussion

Clinicians in resource-limited countries often make decisions to initiate TB treatment in SSN TB suspects based on clinical predictors subject to individual physician discretion. In this study, we found that the predictive factors for a clinical decision to initiate TB treatment in a SSN HIV-infected patient were abnormal CXR and advanced HIV clinical stage in an urban clinic. These findings corroborate data from Tanzania [24] in which an abnormal CXR in the absence of symptoms was the most common basis for the suspicion of TB. Some studies suggest that abnormal CXR is less useful in TB diagnosis in HIV-infected patients [24], [25], [26], [27]. However, others have found CXR to be important in TB screening and diagnosis [28], [29]; this investigation remains an important factor influencing decisions to empirically treat for TB in SSN HIV-infected patients, as was seen in our study.

Many studies have reported increasing prevalence of SSN TB [8], [10], [13] and mortality [1], [2], [3], [4] in advanced immunosuppression and this may explain the association between empiric treatment and advanced HIV as was shown in our study. Furthermore, patients with advanced HIV are often very sick and waiting for further investigations causes delays which may result into death [1], [10].

Our study also established that more than one-third of SSN HIV-infected TB suspects were empirically initiated on TB treatment based on clinical judgment in this outpatient clinic and only 35.3% of these had microbiologically-confirmed TB. These findings agree with those of an earlier Tanzanian study [24] in which only 28% of those who had been presumptively treated for TB had microbiologically confirmed TB. The sensitivity and specificity of clinical judgment in SSN TB diagnosis was 62.5% and 73.7%, respectively. The low accuracy findings in our study could be explained by other co-infections or clinical symptoms mimicking TB, common in HIV-infected patients which could reduce the accuracy of clinical diagnosis. In addition, HIV-infected TB suspects may be more likely to have disseminated TB that may not be able to be diagnosed by sputum culture.

Our findings expand previous findings regarding accuracy of clinical diagnosis of SSN TB in RLS. An earlier Ugandan study [25] among hospitalized patients found the sensitivity and specificity of clinical judgment of 37% and 81% respectively. The higher sensitivity in our study compared to the latter may be explained by the differences in the study populations; the hospitalized patients in the earlier study are likely to be more ill with increased likelihood of co-morbid disease. The lower specificity in our study implies that clinicians in this integrated TB-HIV clinic more commonly empirically initiate TB treatment in HIV-infected TB suspects whose sputum smears are negative. This could be because the clinicians are avoiding the consequences of diagnosis and treatment delays in SSN TB that are emphasized in most TB/HIV settings [30], [31]. Studies have shown significant mortality [30], [31] and high incidence of TB [32] in non-treated SSN TB suspects.

The advent of GeneXpert MTB/rif, an automated nucleic acid amplification test, can decrease the time to diagnosis compared to culture-based methods that may take many weeks to establish a microbiologic diagnosis of TB in SSN patients [33], [34]. However, early data from the roll-out suggests that although some penetration of the equipment into urban and reference centers has occurred, its adoption in more rural areas has been limited by the need for physical infrastructure and the cost of the test [35]. Recent evaluations in India, South Africa and Uganda showed that GeneXpert MTB/rif was cost-effective due to its ability to substantially increase case finding and thereby avert morbidity and mortality [36]. The urine lipoarabinomannan test may also be useful in the diagnosis of SSN TB in severely immunosuppressed HIV-infected individuals and may offer a useful adjunct in this patient population [37], [38]. Recent evaluations of the lateral flow platform show that it may be most sensitive in HIV patients with CD4 cell counts <100 cells/µl [37], [38]. These tests will help to avoid missing diagnosis of patients who have atypical presentations and are not considered for therapy.

The study had some limitations. First, we studied an outpatient HIV population and these results may not be generalizable to hospitalized patients in RLS with high TB/HIV prevalence. Furthermore, the information on the clinical decision to initiate TB treatment or not was obtained retrospectively from clinic records which may not be as complete as required and the predictive factors reviewed here may not be exhaustive. Clinical symptoms were not reported in some of the study patients and this may have diminished the study’s ability to find other significant predictive factors, although we did perform a sensitivity analysis excluding those patients who did not have complete clinical information and the results did not change. Furthermore, we cannot ascertain if all culture negative patients who were empirically treated for TB did not actually have active TB disease since current methods often fail to confirm disease in some subjects [11]. However, confirmation of TB diagnosis was maximized by performing both liquid and solid cultures on the same samples. Finally, we did not assess the effect of TB treatment delay on patient outcome in participants whose TB treatment was initiated later on after culture results were available. We also did not assess the study patients’ response to TB therapy.

Our findings reflect the reality of making a diagnosis of SSN TB in high HIV/TB prevalence settings which may have important implications for HIV/TB management in RLS. From our findings, it is clear that although it is cheap and easy to use the easily available clinical and radiological characteristics to clinically diagnose TB, these are not reliable. This highlights the need for accurate, rapid and affordable point of care tests for diagnosis of SSN TB as well as methods to improve the sensitivity of smear microscopy in order to avoid missed diagnoses in culture-positive individuals.
